# Cell Proliferation Indices in Regenerating *Alitta virens* (Annelida, Errantia)

**DOI:** 10.3390/cells12101354

**Published:** 2023-05-10

**Authors:** Alexandra Y. Shalaeva, Vitaly V. Kozin

**Affiliations:** Department of Embryology, St. Petersburg State University, 199034 St. Petersburg, Russia; a.shalaeva@spbu.ru

**Keywords:** proliferation, cell kinetics, cumulative labeling, quantitative analysis, blastema, regeneration, invertebrates, annelids

## Abstract

In recent years, interest in the possible molecular regulators of cell proliferation and differentiation in a wide range of regeneration models has grown significantly, but the cell kinetics of this process remain largely a mystery. Here we try to elucidate the cellular aspects of regeneration by EdU incorporation in intact and posteriorly amputated annelid *Alitta virens* using quantitative analysis. We found that the main mechanism of blastema formation in *A. virens* is local dedifferentiation; mitotically active cells of intact segments do not significantly contribute to the blastemal cellular sources. Amputation-induced proliferation occurred predominantly within the epidermal and intestinal epithelium, as well as wound-adjacent muscle fibers, where clusters of cells at the same stage of the cell cycle were found. The resulting regenerative bud had zones of high proliferative activity and consisted of a heterogeneous population of cells that differed in their anterior–posterior positions and in their cell cycle parameters. The data presented allowed for the quantification of cell proliferation in the context of annelid regeneration for the first time. Regenerative cells showed an unprecedentedly high cycle rate and an exceptionally large growth fraction, making this regeneration model especially valuable for studying coordinated cell cycle entry in vivo in response to injury.

## 1. Introduction

The regeneration of organs and body parts is a remarkable phenomenon involving changes in cell fate and proliferative status. The study of regeneration on organismal models has given a comprehensive understanding of the complexity of this process and has great potential for determining the fundamental mechanisms of cellular plasticity. Epimorphic regeneration includes several stages, which are wound closure, formation of the wound epithelium, induction of the blastema, and its growth, patterning, and differentiation, followed by functional restoration of the lost structure [1,2,3]. As one of the crucial elements of this process, the regeneration blastema consists of undifferentiated cells undergoing active mitotic divisions, thus providing cellular material for subsequent stages. Even in representatives of the same phylum, for example, in annelids, the cellular sources of regeneration can vary significantly. Depending on the organism species, the source of blastemal cells may be stem cells migrating from distant segments [4,5,6,7] or dedifferentiated cells from the wound-adjacent tissues [1,8,9,10].

Long-distance cell migrations to the wound site in invertebrates are described mostly for planarians and oligochaete annelids. These stem cell populations are referred to as neoblasts. In planarians, neoblasts are distributed throughout the body’s parenchyma and comprise almost 20% of the total number of cells. It is the only proliferating cell population that produces lost body parts after wounding [11]. Neoblast specialization takes place during different phases of the cell cycle and is most likely a labile and transient state [12]. After wounding, neoblasts initiate a missing-tissue response, interpreting positional information. This information, coming from muscles expressing position-control genes, is restored through wound signaling components (*wnt* and *notum*). Positional information determines stem cell fates, while local organizers promote blastema formation [13]. Neoblasts of oligochaetes have different mechanism of action. Randolph, in 1892, first described huge cells in the regeneration of *Lumbriculus*, and she called those cells neoblasts [4]. Neoblasts of oligochaetes migrate from their initial septum-adjacent site towards the wound along the ventral nerve cord [14]. In enchytraeid *Enchytraeus japonensis*, pulse-chase BrdU labeling uncovered that neoblasts ensure the formation of the mesodermal part of blastema, but epidermal and gut tissues have distinct proliferation patterns and seem to be restored from the local dedifferentiation of those tissues [6].

Local cell dedifferentiation in annelids was shown for muscle cells in *Owenia fusiformis.* Using electron microscopy, Fontés and colleagues [15] tracked how muscle cells undergo drastic changes in morphology. However, the muscle-derived identity of blastemal cells was proved by molecular markers [15]. Double-labeling by BrdU and EdU on *Syllis malaquini* showed that the blastema consisted of cells that incorporated the thymidine analogs after amputation, meaning that the main source of the blastema is local cell dedifferentiation [10].

Blastemal cells, despite their origin, are actively dividing, gaining the necessary cellular mass to differentiate and form the lost structures. Stages prior to blastema formation are often independent of active proliferation [16,17,18]; however, it becomes crucial later on [2,9]. Proliferation of the involved cells must be strictly controlled for the successful reparation of the lost part. The cell cycle has several checkpoints between the phases, which, if passed successfully, allow the cell to transfer to the next stage [19,20]. The source of the signal stimulating cell divisions and blastema initiation may be the damaged nerve fibers or the wound epithelium [21,22,23,24,25]. Different signaling pathways play significant roles in regeneration; however, a direct link between the growth factors and proliferation has been confirmed mostly for vertebrates [26,27,28,29,30,31], as well as for the annelid *Alitta virens* [32]. Transcriptome analysis of annelid regeneration uncovered that some factors of signaling pathways, which control proliferation, such as Wnt/β-catenin [33,34] or the FGF pathway, are up-regulated, which makes them an interesting candidate for further research [2,32].

The quantitative analysis of proliferating cells and changes in the cell cycle length has made a huge contribution to the understanding of tissue homeostasis, but it has rarely been applied other than to classical animal models [35,36,37,38]. Since the description of cell cycle analysis methods based on the radioactive 3H-thymidine isotope labeling of nuclei in the S-phase [39], new ways to assess proliferation have emerged using modified DNA precursors, BrdU, EdU, and other halogenated tags. Different tags in different experiment types opened a field for not only proliferation analysis but also for cell cycle evaluation as well as proliferative fractions in selected tissue [40].

The regeneration of *A. virens* has been greatly described at the molecular level [32,41,42,43], but the cellular kinetics of the process have not been investigated in detail. So far, neither in the nereid polychaete *Platyneresis dumerilii* nor in other marine annelids, such as *S. malaquini* [10], *Dorvillea bermudensis* [44], and *Capitella teleta* [8], oligochaetes *Enchytraeus japonensis* [6], or *Lumbriculus* [45], has cellular proliferation been assessed quantitatively. 

**Figure 1:**
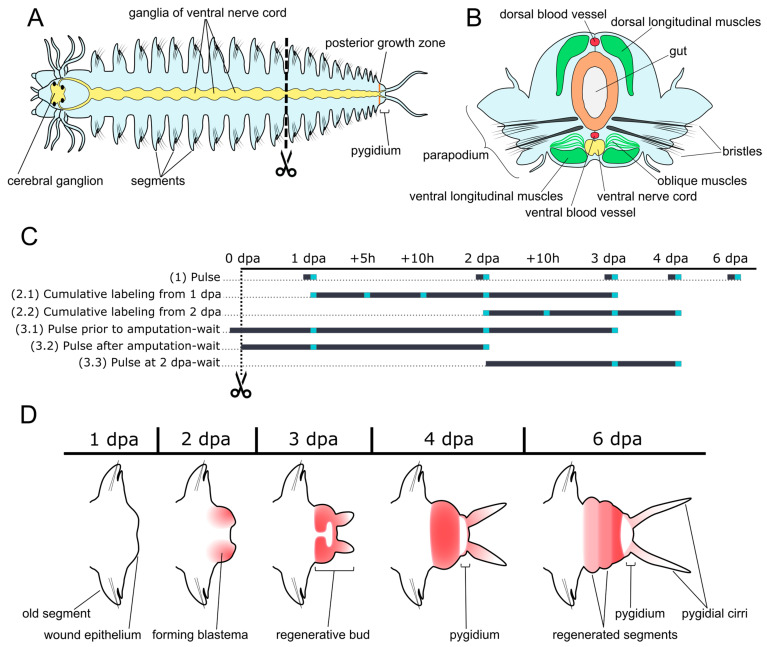
Schematic representation of *A. virens* general anatomy (**A**,**B**), posterior regeneration (**D**), and experimental design (**C**). (**A**) *A. virens* body plan, including the central nervous system (yellow). Anterior end of the body is to the left, vertical dotted line indicates an amputation plane. (**B**) Transverse section of the adult body segment, demonstrating muscle fibers (green), ventral nerve cord (yellow), blood vessels (red), the gut wall (brown), and bristles (black rods). (**C**) Experiment schemes (in lines). Numbers in parenthesis indicate the experiment type: (1) pulse labeling, (2) cumulative labeling, (3) pulse-wait labeling. Gray lines indicate periods of EdU labeling. The gray gradient in type 3 experiments reflects dilution of the incorporated EdU label. The cyan squares are the time points of sampling. (**D**) Schemes of anatomical restoration of the posterior body end (directed to the right) at the first 6 days post-amputation (dpa). Zones of proliferative activity recovered by the pulse-EdU labeling are indicated in red (color intensity reflects the density of labeled nuclei).

*A. virens* has a complex segmented body plan (Figure 1A,B) and can regenerate the posterior end of the body. This process occurs rather quickly: within seven days, a juvenile worm is able to regenerate several new segments [46], which makes it a convenient model for regeneration research. Here we describe the proliferation patterns during the posterior segments’ regeneration of *A. virens*, focusing on its spatial, temporal, and quantitative parameters. We analyzed the cell proliferation quantitatively, first using the labeling index (LI), and found the proportion of S-phase nuclei to the total number of nuclei. These indices helped us in the estimation of the cell cycle parameters, such as the length (Tc) and the length of the S-phase (Ts), as well as in the calculation of the growth fraction (GF). Our research highlights cellular heterogeneity in the blastema and rapid cell cycle alterations already at the early stages of regeneration. Altogether, our data provide insight into cell cycle kinetics that can be used for further comparative investigation of the cellular aspects of animal regeneration.

## 2. Materials and Methods

### 2.1. Animals

Spawning epitoke individuals of *A. virens* were caught in the summer near the Marine Biological Station of SPbSU in the White Sea. A laboratory culture of embryos was obtained by artificial fertilization [47]. The animals grew for 2–3 months in small aquariums with natural or artificial seawater until they reached 15–20 segments in length. The posterior thirds of the juveniles’ bodies were amputated, and then the animals were left to regenerate for various time periods at +18 °C. At the preferred stages (1–6 days post-amputation, dpa), the specimens were anesthetized with 7.5% MgCl_2_ mixed with artificial seawater (1:1) and fixed in 4% paraformaldehyde on 1.75× PBS with 0.1% Tween-20 overnight at +4 °C. The samples were washed two times and stored in 100% MetOH at −20 °C.

### 2.2. EdU Incorporation and Detection

We performed various experiments on 5-ethynyl-2-deoxyuridine (EdU, a thymidine analog) labeling (Figure 1C) and aimed to estimate some aspects of proliferation and the cell cycle (length of the S-phase (Ts), cell cycle length (Tc), and growth fraction (GF)) in *A. virens*. We used 1 mL of 5 µM EdU diluted in artificial seawater for the incubation of one worm for 15 min (experiments (1) “pulse”, and (3.1), (3.3) “pulse-wait”), 1 h (experiments (3.2), (3.4) “pulse-wait”) or up to 48 h (experiments (2.1), (2.2) “cumulative labeling”). In the latter case, the EdU solution was changed every day. Experiment (3.4) was performed on intact non-amputated worms that were incubated in EdU and washed and fixed 1, 2, and 3 days after labeling. All other experiments were carried out on regenerating worms according to the scheme depicted in Figure 1C. EdU labels cells that are in the S-phase at the time of incubation. Upon mitotic division, the label transfers to the daughter cells, allowing for the visualization of the fate and location of the descendant cells as well. After labeling in dark conditions, the specimens were either fixed as described above or washed 5 times in 5 mL of seawater per specimen and kept in the dark until fixation. To detect EdU labeling, we used the “click” reaction [48,49]. The “click” reaction involves fluorescent azide–alkyne cycloaddition catalyzed by Cu(I). Before the “click” reaction, we rinsed samples in 0.1 M TRIS buffer (pH = 8.5). The reaction mix included 100 mM TRIS (pH = 8.5), 4 mM CuSO_4_, 2 µM sulfo-cyanin-5-azide, 50 mM ascorbic acid, and deionized water. Incubation for 45 min in the reaction mix was followed by washes in TRIS buffer (pH = 7.4) and nuclear DNA staining in DAPI (1 µg/mL). The samples were mounted in 90% glycerol for visualization. The sample size varied from 4 specimens per experiment up to 16 specimens (see Supplementary File S1, the “sample size” tab, for details on each experiment).

### 2.3. Visualization and Cell Counting

Confocal images were obtained using the Leica TCS SPE confocal microscope. We used a 40× lens with a 1.5 µm step between planes. For the statistical analysis, we evaluated the first 45 planes of the regenerative bud in each specimen, which made up almost half of its depth in most cases, and sometimes even more. All specimens were scanned from the ventral side. Optical sections were combined in stacks by ImageJ. Schemes were made in Adobe Illustrator and Inkscape.

For experiment types 1 and 2, we calculated the number of labeled cells within the regenerative bud in confocal Z-stack using Bitplane Imaris 7.5 software. We manually specified the region of interest (regenerative bud) and separated it using the “Surfaces” tool. Then we estimated the relative size of several nuclei using the “Slice” function. An average value of the nuclear diameter was specified using the “Spots” tool. After automated quantification of the objects, we manually adjusted the threshold level so that all nuclei were counted, and the signal/false positive ratio was adequate. The results of the first automatic calculations were verified by manual counting of the nuclei in ImageJ using the Cell Counter plugin. After registration of the EdU+ and DAPI+ nuclei numbers, we calculated the labeling index (LI), which is a ratio of cells in the S-phase to the total amount of cells multiplied by 100. Statistical analysis was performed in MS Excel, Past, and R. For each quantified sample, we calculated the mean values and standard errors (Supplementary File S1). The obtained values of LI and the total amount of registered cells were examined using one-way Kruskal–Wallis tests and Mann–Whitney pairwise post-hoc tests.

### 2.4. Cell Cycle Parameters

The cell cycle parameters in the regenerating tissue were assessed by the cumulative labeling method in experiments (2.1) and (2.2) at the stages of 1 to 3 dpa and 2 to 4 dpa, respectively. We incubated the regenerating worms in EdU solution for up to 48 h and fixed them after 15 min, 5, 10, 24, and 48 h of exposition. After EdU detection, we determined and plotted the LIs of each specimen at a certain time point, and fitted a cumulative curve for the obtained values. To evaluate the cell cycle parameters based on the cumulative curve that reached a plateau, we used a method described by Nowakowski and colleagues [50]. The cumulative curve is described by the equation: y = a + bx, where “a” is the intercept, “b” is the slope, meaning that before the break point, we observed a linear regression, then at the break point, the regression reached a plateau. After visualizing the cumulative curves in R by fitting them with the least squares method (with packages “nlraa” and “minpack.lm”), we estimated the approximate cell cycle parameters, such as the growth fraction (GF), the length of the S-phase (Ts), and the length of the cell cycle (Tc). The growth fraction was found by the plateau value of the curve when all dividing cells had an EdU label and the amount did not increase any more. The break point of the curve corresponded to the time Tc-Ts, so that by extrapolating a regression line to the *y*-axis, we found the Ts. Knowing the Ts, we could estimate the Tc by adding the Ts to the break point value. All of these parameters are described by equations from the mentioned model [50]:

f(t) = GF × (t + Ts)/Tc, for t ≤ Tc − Ts,

and

f(t) = GF, for t ≥ Tc − Ts.

## 3. Results

### 3.1. Pulse Labeling

**Figure 2:**
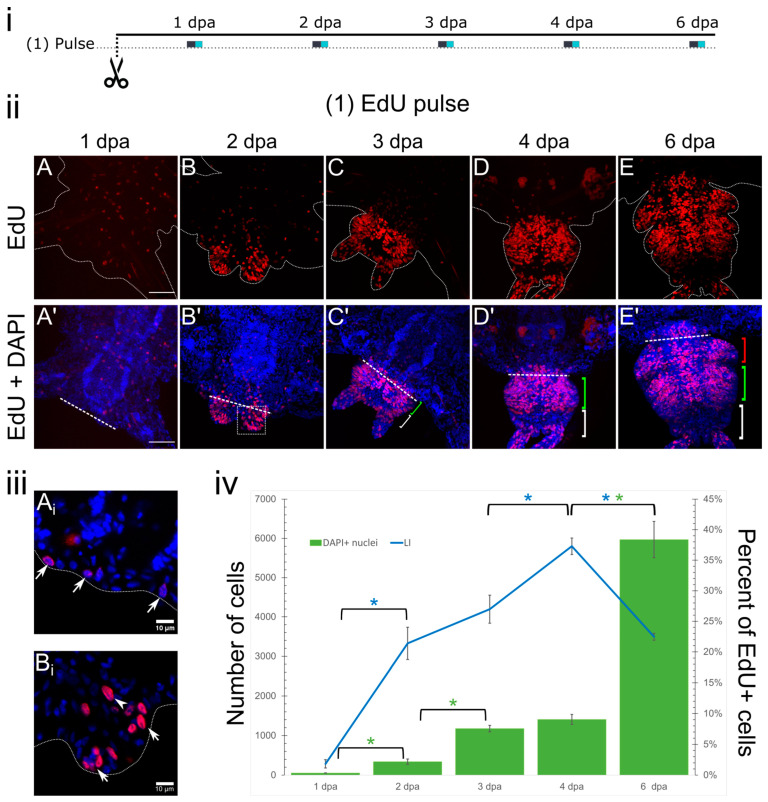
Pulse-labeling results in posterior regeneration of *A. virens*. (**i**) Experimental design of the pulse labeling; cyan squares indicate time points when samples were collected, grey lines are periods of EdU incubation. (**ii**) Maximum projections of Z-stacks scanned from ventral side of the body. EdU (red channel) and DAPI (blue channel) visualization in representative specimens at the stages of 1 (**A**), 2 (**B**), 3 (**C**), 4 (**D**), and 6 dpa (**E**). Anterior is up or up-right, dotted lines in (**A**–**E**), (**A_i_**,**B_i_**) are outlines of the body; pygidium is marked by a white bracket, green bracket indicates an area of future segmental tissues, red bracket indicates the first newly formed segment, straight line in (**A’**–**E’**) indicates the amputation site. (**A**,**A’**–**E**,**E’**) Scale bar is 50 µm. (**iii**) Individual planes of confocal Z-stack at 1 dpa stage (**A_i_**) or 2 dpa (**B_i_**), where arrows point to proliferating epithelial cells and arrowhead points to proliferating blastemal cells. (**A_i_**,**B_i_**) Scale bar is 10 µm. (**iv**) Labeling index and cell count values during the first 6 days of regeneration. Error bars indicate standard error of the mean (SEM); asterisks above brackets indicate statistically significant differences; *p*-value ≤ 0.005 for the Mann-Whitney test (blue asterisks for LI, green asterisks for number of cells).

In the type (1) experiments, we identified the zones of proliferative activity in the regenerating juveniles by short EdU labeling (Figure 1D). We also estimated the proportion of simultaneously proliferating cells (the labeling index, LI) in the regenerative bud. At the 1 dpa stage, only sparse individual labeled nuclei were present in the wound epithelium (Figure 2A,A’,A_i_), and the LI at this stage was unsurprisingly low 1.8 ± 0.7% (Figure 2iv). EdU-positive nuclei were also found in the ventral nerve cord and in longitudinal muscles; however, the location of these nuclei lacked any anterior–posterior gradient, indicating the absence of an obvious wounding response at this stage. Starting from 2 dpa, and over subsequent regeneration stages, EdU incorporation within the wounded segment became more prominent near the amputation site compared to the anterior part of the same segment. Gut cells there had higher EdU incorporation rates; however, some coelomic and epidermal cells were also EdU-positive.

By the 2 dpa stage, most of the nuclei in the regenerative bud were in the S-phase, which made the labeling more extensive (Figure 2B,B’). EdU-positive nuclei were predominantly found in the lateral domains of the epidermis and blastemal cells (Figure 2B_i_), which differ from wound epithelium cells by their elongated nucleus shape and size. At this stage, the LI drastically increased and reached 22.8 ± 3.6% (Figure 2iv). By the 3–4 dpa stage, the regenerative bud becomes more pronounced, the pygidium forms posteriorly, and resegmentation events take place [46]. EdU incorporation is more prominent at the segment formation area compared to the pygidium region and cirri, where proliferation is less active (Figure 2C,D). In the regenerative bud, EdU-positive cells were found in the epithelium, newly formed gut, and coelomic sacs, which, by this stage, were reemerging.

At 4 dpa, the LI reached a maximum value of 37.3 ± 1.35%. The number of registered cells by the 4 dpa stage also increased four-fold compared to the 2 dpa stage and reached 1408 ± 124 cells (Figure 2iv). By the 6 dpa stage, the most active proliferation was also observed in the developing segmental tissues; however, the more mature segment in the anterior part of the bud seemed to be less proliferatively active (Figure 2E’, red bracket). The LI at this stage decreased to 22.5 ± 0.6%; however, the number of registered cells was the highest (5970 ± 463) (Figure 2iv). Comparison of the proliferation values at successive time points demonstrated that statistically significant increases in the LI (at 2, 4, and 6 dpa) were always followed by a significant increase in the cell number at the next sampled stage (at 2, 3, and 6 dpa). This confirms EdU labeling as a marker of mitotic activity in our model.

### 3.2. Cell Kinetics in Cumulative Labeling

**Figure 3:**
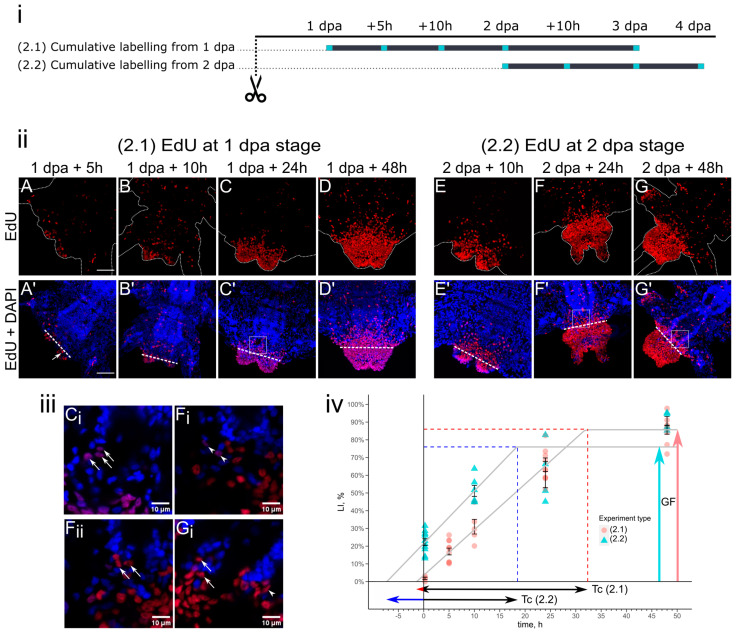
Cumulative labeling starting at 1 dpa (2.1) or 2 dpa (2.2). (**i**) Experimental design; cyan squares indicate time points when samples were collected, grey lines are periods of EdU treatment. (**ii**) Maximum projections of confocal Z-stacks scanned from ventral side demonstrate dynamics of EdU incorporation (red channel), and DAPI nuclear staining (blue channel). Anterior is up or up-right, dotted line in (**A**–**G**) is outline of the body, straight line in (**A’**–**G’**) indicates the amputation site. Scale bar is 50 µm. Arrow in (**A’**) points to the first noticeable blastemal cell. (**iii**) Single optical planes of zoomed regions outlined by square in **C’** (**C_i_**), **F’** (**F_i_**,**F_ii_**), **G’** (**G_i_**). Arrows point to proliferating cells in ventral nerve cord, arrowheads indicate labeled nuclei in muscles, scale bar is 10 µm. (**iv**) Cumulative labeling curves with labeling indices in experiment (2.1) (red circles) and experiment (2.2) (blue triangles). Dotted lines are projections on axes from the breakpoints of curves and indicate time periods of (Tc–Ts) and percentages of GF (also shown by vertical arrows on the right). Error bars represent SEM. Double-sided arrows are lengths of cell cycle (Tc) for different experiments, colored parts are lengths of Ts. In experiment (2.1), Tc was 33.2 h and Ts was approximately 1.3 h, GF equaled 85.7%; in experiment (2.2), Tc was 25.7 h, Ts was 7.3 h, GF equaled 76%.

In the type (2) experiments, with constant exposure to EdU, we observed a continuous increase in the LI until the cumulative curve reached a plateau (Figure 3). In experiment (2.1), at the 1 dpa stage, individual labeled nuclei were observed in the wound epithelium. After 5 h of EdU incorporation at the 1 dpa + 5 h stage, proliferating cells were noticed in the epithelium, ventral nerve cord, gut, and in other internal mesodermal tissues in the wounded segment. As for the wounding site, labeling was predominantly noticed in the epithelial cells and in the first blastemal cells (Figure 3A’, arrow). Over the next 5 h (1 dpa + 10 h), the labeling was enriched and present in the same domains as previously described (Figure 3B). After 24 h of EdU incorporation, most of the proliferating cells were localized in the epithelium of the regenerative bud (Figure 3C). At this point, the labeling index increased by almost three-fold (from 26.2 ± 5.8% to 64.9 ± 2.8%, (Figure 3iv, red circles)). In the old segment, most of the EdU-labeled nuclei were localized near the wounding site, and most of them were in the superficial epithelium and ventral nerve cord. By 48 h of incubation, the number of EdU-positive nuclei in the regenerative bud reached 85.7 ± 2.5%, indicating an unprecedentedly high GF (Figure 3iv). By this time (1 dpa + 48 h), almost all epidermal cells of the regenerative bud were labeled, which was not the case for the blastemal cells. In the neighboring old segments, a proliferation marker was present in the same manner as after 24 h of incubation, although its level was obviously increased (Figure 3D).

In experiment (2.2), at 2 dpa, in the specimens after 10 h of EdU incorporation, the labeling in the blastemal cells was quite prominent (Figure 3E) and its overall localization was similar to the 15 min of pulse labeling (Figure 2B). In the adjacent segment, labeled cells were localized predominantly in the ventral epithelium near the wounding site (Figure 3E’). At further stages of regeneration, this tendency remained the same, and most of the EdU-positive cells were found in the epithelium closest to the bud–segment border (Figure 3F,G). During those stages (2 dpa + 24 h/48 h), the EdU label distribution was similar, although in the latter stage (2 dpa + 48 h), the proportion of EdU-positive nuclei was higher. Accordingly, the labeling index notably changed from 61.4 ± 8.4% at 2 dpa + 24 h to 90.6 ± 2.6% at the 2 dpa + 48 h stage (Figure 3iv, blue triangles). EdU-positive nuclei were found in the nervous system both in the wounded (Figure 3F_i_,G_i_, arrows) and unwounded ganglia of the adjacent segments (Figure 3F’,G’) and in the muscle fibers (Figure 3F_i_,G_i_, arrowhead).

The increase in the LI value (the cumulative curve slope) reflects that the rate of entry into the S-phase was higher in experiment (2.2) compared to (2.1). During the first 24 h of experiment (2.1), the LI drastically increased from 1.8 ± 0.7% (after 15 min of incubation) to 17 ± 1.9% (at 1 dpa + 5 h). The next increase occurred from 10 h (30.1 ± 4.1%) to 24 h (64.9 ± 2.8%), and by 48 h, the LI reached 85.7 ± 2.5%. Using these labeling indices, we plotted a cumulative curve (Figure 3iv). From the graph of (2.1), we estimated the growth fraction, which was 85.7%, the approximate length of the S-phase was 1.3 h, and the overall cell cycle length was approximately 33.2 h, which equals the sum of the breakpoint time and the Ts length (31.9 h + 1.3 h). In experiment (2.2), the LI initial value was 22.3 ± 1.9% after 15 min of incubation, which reached 51 ± 3.1% after 10 h of incubation, and then only increased by approximately 10% after the next 14 h (by 2 dpa + 24 h the LI equals 61 ± 8.4%). After 48 h of EdU incorporation, the LI was 90.6 ± 2.56%. For cumulative labeling after the 2 dpa stage in (2.2), cell cycle parameters differed compared to the (2.1) values. These changes were multidirectional due to the shortening of the overall cell cycle length, which was 25.7 h and contrasts the Ts longer time, which was approximately 7.3 h, and the decreased growth fraction, which was equal to 76% (Figure 3iv).

### 3.3. Pulse-Wait Labeling

**Figure 4:**
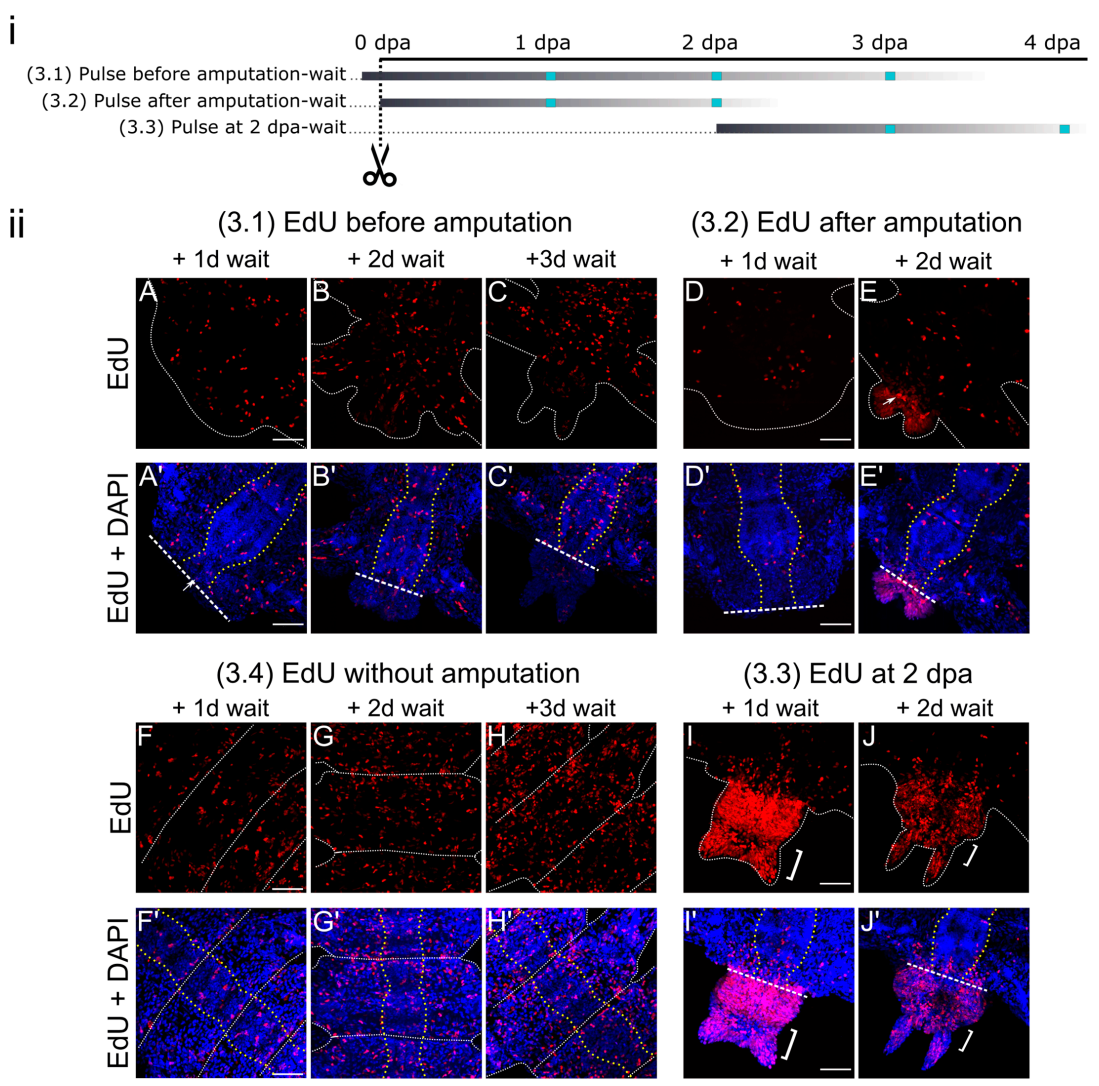
Pulse-wait labeling starting before amputation (3.1), immediately after amputation (3.2), at 2 dpa (3.3), and in intact non-amputated specimens (3.4). (**i**) Experimental design; cyan squares indicate time points when samples were collected, grey lines are periods of EdU incorporation (at the left end) and dilution of the label due to mitotic divisions over time (decreasing gradient to the right). (**ii**) Maximum projections of confocal Z-stacks scanned from ventral side demonstrate dynamics of EdU labeling (red channel), and DAPI nuclear staining (blue channel). Anterior is up or up-right, white dotted line is an outline of the body in (**A**–**E**,**I**–**J**), and outline of segment borders in (**F**–**H**), straight line in (**A’**–**J’**) indicates the amputation site, pygidium is marked by a white bracket, yellow dotted lines in (**A’**–**J’**) indicate an outline of the ventral nerve cord. Arrow in (**A’**) points to labeled nucleus of muscle fiber, arrow in (**E**) points to intensively labeled nucleus of blastemal cell. Scale bar is 50 µm.

We performed label-retaining assays (3.1–3.4) to evaluate the input of the wound-adjacent segments in the regenerative bud formation and the impact of wounding on proliferation (Figure 4). After short EdU incorporation, samples were rinsed and left for regeneration up to 3 days. A similar experimental scheme was performed on non-regenerating juvenile worms. Experiment (3.4) demonstrated the baseline proliferation in normal physiological conditions (Figure 4F–H). Incubation in EdU before and immediately after the amputation labeled cells that were mitotically active at the moment of labeling. Later on, during the waiting time, those cells retained the label and may have contributed to blastema formation.

In intact worms (3.4), we evaluated the cellular distribution in segments of the posterior third of the body where we normally would have carried out the amputation. The overall number of cells there became visually more prominent at the successive time points (Figure 4F–H). Due to the dilution of the EdU label among the daughter cells, we suggest that differentiated cells undergo mitotic divisions during the normal physiological state, when juveniles are constantly growing and increasing their segment numbers. Proliferating cells were found in the gut and mesodermal tissues, such as muscles and coelomic cells. Within the ectodermal and neural derivatives, cell proliferation seemed to be less prominent, compared to other tissues, but nonetheless detectable. The labeled cells were forming “clusters” already after 1 day of waiting (Figure 4F), indicating that local proliferation was more prominent in specific parts of the same tissue.

Incubation in EdU prior to amputation (3.1) had comparable results with experiment (3.4) regarding the proliferation pattern in the segmental tissues (Figure 4A–C). Individual EdU-positive nuclei and their couples in muscle fibers (Figure 4A’, arrow) and in the ventral nerve cord on the wound-adjacent side were observed at 1 dpa (Figure 4A). On the second day of regeneration, diluted labels were found in individual blastemal cells (Figure 4B’). The observed number of EdU-positive nuclei in the regenerative bud was visibly lower compared to the overall 2 dpa proliferation detected by the pulse labeling (Figure 2B). Those faintly labeled cells, being daughters of mitotically active cells in intact animals, can be found predominantly at the gut–epidermal border. A similar labeling pattern in the growing tissues of the regenerative bud was registered at the 3 dpa stage (Figure 4C). As for wound-adjacent segments, starting at the 1 dpa stage and further on, most proliferating cells were found in the gut epithelium. Mesodermal tissues, ventral nerve cord, and superficial epithelium were actively proliferating not only at the amputation site, but in the entire segment. The labeling patterns in those segments were similar to those in the non-amputated samples (3.4) tissue-wise and regarding the dilution of the EdU signal. This might indicate that cells undergoing mitosis before amputation have minimal impact on blastema formation. Thus, amputation might influence the mitotic activity that is necessary for the accumulation of reparation-responsible cells.

To check this assumption, we incubated juveniles in EdU immediately after amputation in experiment (3.2). During the first 24 h after amputation, there were only a few proliferating cells in the epithelium, ventral nerve cord, mesodermal tissues, and gut (Figure 4D), which contrasts the broader labeling of the segmental tissues in experiments (3.1) (Figure 4A) and (3.4) (Figure 4F). However, it was obvious that the first divisions in the samples (3.2) already occurred, since we observed couples of cells near each other. After the next 24 h of waiting, the segmental tissue labeling was slightly wider (Figure 4E); however, it contrasted even more with experiments (3.1) (Figure 4B) and (3.4) (Figure 4G). In the regenerative bud, we observed diluted EdU labels (Figure 4E), which were present in much more cells than in experiment (3.1) (Figure 4B). Some blastemal cells had much brighter labels than others (Figure 4E, arrow), suggesting their slower mitotic rate. Comparison of the proliferation profiles in experiments (3.1), (3.2), and (3.4) suggests that the mitotically active cells responsible for body growth were different from the cellular sources of regeneration that entered the cycle in response to amputation.

Additionally, experiment (3.3) was aimed at identifying the input of blastemal cells labeled at 2 dpa into new segments. Mitotic cells incorporated the EdU, and later, we observed diluted or finely dispersed labels in the nuclei as an indicator of those daughter cells. At the 3 dpa stage (2 dpa + 24 h wait), the localization of EdU-positive nuclei (Figure 4I) resembled the pulse experiment results (Figure 2C). The regenerative bud consisted of two visible zones that differed in their proliferation pattern, since, in the posterior part (pygidium), the EdU labeling was less intense (Figure 4I, white bracket) than in the anterior (segment-producing) part. The less intense pulse labeling in the pygidium at 3 dpa (Figure 2C,C’, white bracket) compared to the (3.2) sample (Figure 4I, white bracket) indicates a differential decrease in the rate of cell cycle entry in different parts of the regenerative bud. In the wound-adjacent segment, EdU labels were present predominantly in the gut tissues; however, individual EdU-positive cells were found in the superficial epithelium, mesodermal derivatives, and ventral nerve cord. Waiting for 48 h resulted in even more diluted EdU labeling (Figure 4J), indicating that the blastemal and epithelial cells of the regenerative bud continued to proliferate until 4 dpa, which is consistent with the LI dynamics (Figure 2iv).

## 4. Discussion

### 4.1. Spatial and Temporal Dynamics of EdU Incorporation in Regenerative Bud

Posterior restoration of the *A. virens* body is accompanied by extensive proliferation of cells in the regenerative bud as well as localized proliferation within the old segments (Figure 1D and Figure 2). The entire process of regeneration takes from 6 to 9 days (depending on the environmental conditions) and undergoes the standard stages of regeneration [2]. During the first 24 h, wound closure through muscle contraction and formation of the tissue plug takes place, and later, wound epithelium forms at the amputation site. Wound epithelium is mitotically inactive (except for rarely found S-phase nuclei) due to its origin from the intestinal and epidermal epithelial cells closest to the wound, which simply fuse together, forming this transient layer of dedifferentiating cells [1,2,46]. Adjacent tissues of the wounded and unwounded segments did not demonstrate a proliferative response to the amputation at this early stage (Figure 2A and Figure 4A). As the regenerative bud grows and develops, the wound epithelium is replaced with regular epithelium, and it becomes much more mitotically active and remains like that until the first differentiated structures (pygidium and anal cirri) are restored at the terminal posterior body end.

On the second dpa, the regeneration blastema (i.e., the internal mesodermal cells of the early bud) is formed in *A. virens*. It actively incorporates EdU, as well as the epithelium above it (Figure 2B). Closely examining the EdU-positive nuclei distribution, we found that, already at this stage, the bilateral terminal region of the posterior body end seemed to be more proliferatively active, indicating that even before terminal structures and the newly formed segment can be visualized, they are distinguishable in terms of the intensity of their cell cycle entry. Interestingly, some molecular markers found in *P. dumerilii*, such as *Pdum-dlx* (a marker of appendage formation), *Pdum-en*, and *Pdum-wnt1* (some of the early indicators of segmentation), tend to show similar patterns of expression at this stage of regeneration [9].

At the 3–4 dpa stages in *A. virens,* terminal structures acquired lower proliferative activity, but cells from the old segment side seemed to incorporate EdU more intensively (Figure 2C,D). At the 3 dpa stage, the pygidium region with pygidial cirri became evident, as well as a new segment with coelomic sacs, however, without parapodia [46]. We observed peak levels of LI, 37% at 4 dpa, which correlated with the active morphogenetic processes at this stage. Another important process taking place at this time was the re-emergence of the posterior growth zone as a presumptive ring of cells, and its functional launch. In our work, we rarely can visualize the growth zone as a row of synchronously dividing cells; however, previous work on *A. virens* showed that multipotency markers, such as *Avi-vasa* and *Avi-Piwi1*, first reappeared in the growth zone at the 3 dpa stage, indicating its activity [42]. In nereidid *Perinereis nuntia*, the growth zone cells are characterized by synchronized cell cycle entry and other properties, allowing for resegmentation [51]. *Hox* genes expression patterns in *A. virens* [43,52] and in *P. dumerilii* [53] have also indicated the active processes of axial restoration happening at this stage. Thus, the differential proliferation pattern presented here may be established by the preceding expression of these regulatory genes.

By the 6 dpa stage, cell proliferation in *A. virens* became less active and had an obvious anterior–posterior gradient, with higher EdU incorporation in the posterior segmental tissues adjacent to the growth zone (Figure 2E). From this stage onward, we can infer that the reparative process was complete, and normal posterior growth began. The overall labeling dynamics have similarities with those that have been described for other Errantia polychaetes *D. bermudensis* [44], *S. malaquini* [10], and *P. dumerilii* [9].

### 4.2. Contribution of the Wound-Adjacent Tissues to Formation of the Regenerative Bud

Proliferative activity in the wound-adjacent segment is of particular interest since it reflects the mechanisms of the induction of regeneration sources. Cells of so-called old (but physiologically growing by nature) tissues can either locally dedifferentiate and provide the source of blastemal cells [9,10,15], or their multipotent precursors can migrate to the wound site from the adjacent segment as in *C. teleta* [8] and from other parts of the body as in some oligochaetes [6,45]. Migrating cells do not necessarily have stem cell properties, but several distinct cell types can be determined as they migrate towards the wound site and undergo divisions [7].

We used different types of pulse-wait combinations in our experiments to identify the input of segmental tissues in blastema formation (Figure 4). In EdU incubation, before (3.1) and immediately after amputation (3.2) we saw a proliferative response to wounding and compared it with the proliferation happening under normal conditions (3.4). Our results show that the blastemal cells were predominantly descendants of cells entering the cell cycle after amputation (Figure 4E). The proliferation present before amputation contributed very little to the newly restored parts of the body (Figure 4B,C). On the contrary, most of the EdU-positive cells in the (3.1) samples remained in the old segment’s tissues, demonstrating similar localization and abundance as those registered in the intact animals (3.4) at the same waiting time (Figure 4A vs. Figure 4F, Figure 4B vs. Figure 4G, Figure 4C vs. Figure 4H). However, labeling (3.2) immediately after amputation with the subsequent waiting period showed us EdU-positive nuclei couples in the old segment, which we interpreted to have resulted from divisions (Figure 4D). Compared to the (3.1) samples, there were much fewer label-retaining cells, which indicates an immediate decrease in the proliferation of the segmental tissues in response to amputation. Such drastic differences in the EdU distribution between the (3.1) and (3.2) samples support the hypothesis that *A. virens* restores its segments through the local dedifferentiation of cells, which become mitotically active in response to wounding [42]. There is evidence for dedifferentiation within certain structures, such as epithelium and muscles, in the reparative process across annelids [1,2]. In *A. virens*, dedifferentiation seemed to take place in the longitudinal muscles that we identified by clusters of EdU-positive nuclei located near the amputation site within muscle fibers at the early stages of regeneration (Figure 4A). Muscle dedifferentiation at the early stages of anterior regeneration have been also described in the polychaete *O. fusiformis* [15].

Tissues of the nervous system also seemed to have an impact on *A. virens* regeneration. Nerves from the severed ventral nerve cord project and innervate the regenerative bud [46]. Experiments on EdU pulse and pulse-wait labeling at the stages of 1 and 2 dpa recovered EdU-positive cells in the ventral nerve cord at the wounding site (Figure 3B and Figure 4A–C). However, the labeling patterns do not imply continuity of the old neural tissues and the ganglion-producing cells in the regenerative bud. Recent studies on nereidid regeneration have described the neural expression of certain *Hox* genes [43,53] and FGF pathway components [32] at the early stages of regeneration. A neural hormone regulates regeneration in *P. dumerilii* [54]. Nerves themselves are known to regulate and induce the formation of the regenerative bud, both in annelids [55,56,57,58] and vertebrates [21,23,59,60]. Thus, the regulation of proliferation by the nervous system in annelid regeneration seems to be an important area for future research.

As for the intestine, which appeared to be the most proliferatively active in the majority of our experiments, its cells were the only source of newly formed gut tissues (Figure 4A–C). The same conclusions are valid for *P. dumerilii* [9], *S. malaquini* [10], and *D. bermudensis* [44]. The intestine is likely remodeled by morphallaxis and fused with epidermal epithelium at the wound site [2]. The wound-adjacent intestinal tissues lack dividing cells during the first two days of regeneration. At this time of *A. virens* regeneration, the posterior portion of the intestine launches the expression of *foxA* [41], a marker gene for the ectoderm-derived gut tissues. Thus, the inferred molecular morphallaxis of the gut [41] may be responsible for delayed proliferation in this organ.

### 4.3. Cell Cycle and Subpopulations of Proliferating Cells in Blastema

Through the cumulative labeling approach, we evaluated the approximate parameters of the cell cycle at the early stages of *A. virens* regeneration. The S-phase varied from 1.3 to 7.3 h depending on the experiment type (Figure 3). Similar Ts durations were described in the sponges’ regeneration, but the overall cell cycle duration was significantly shorter [35]. The G2, M, and G1 durations in our experimental setup could not be strictly evaluated, but the overall duration of the cell cycle (Tc) was 33.2 h in the type (2.1) experiment and 25.7 h in (2.2). The growth fraction in the regenerative bud never reached 100%, which means that some cells either had completely different cell kinetics (e.g., synchronously dividing cells of the posterior growth zone [51]), or stopped cycling very early.

Comparing experiments (2.1) and (2.2), which examined cell populations at 1–3 dpa and 2–4 dpa, we noticed an increase in the Ts, but an overall shortening of the Tc (Figure 3iv). Changes in the calculated cell cycle parameters might indicate some undergoing transformation in the cell states, such as moving from the quiescent state to active mitosis, or changes in the composition of a heterogeneous population. A hypothesis of punctuated cycling was initially proposed for newt limb regeneration as an explanation for the different growth rates between adult and larval newts. The size of the quiescent cell population determines the speed, rate, and success of regeneration in newts [61]. It is noteworthy that the size of the G1-G0 cell population increases if the regeneration blastema in the newt limb is denervated [62] indicating that nerves and wound epithelium act as controlling factors of regeneration by influencing the cell cycle [63]. Despite the significant evolutionary distance between the mentioned organismal models, these observations on amphibian regeneration are consistent with our results on *A. virens*, which has made us start looking for factors that slow down the entry into the cell cycle (detected by the decrease of LI from 4 to 6 dpa) and reduce the GF already at 2–4 dpa.

We also noticed heterogeneity in the spatial distribution of the proliferating cells in the *A. virens* regenerative bud. The superficial epithelium and blastema seemed to incorporate EdU at different rates. Specifically, at the end of experiment (2.1), virtually all epithelial cells bore the EdU label (by visual examination), while blastemal cells demonstrated visibly lower mitotic activity (Figure 3). Heterogeneity of the blastemal cells was also confirmed by experiments (3.1) and (3.2), which showed distinct labeling patterns (Figure 4). In addition, the regenerative bud cells in the (3.2) samples had varying intensities of EdU labels (Figure 4E), suggesting their different mitotic rates. The anterior–posterior heterogeneity in the proliferative pattern was also noticed by pulse labeling after the 2 dpa stage in *A. virens*. Altogether, the presented and previously published data allow us to speculate that since the formation of regenerated tissues in *A. virens*, they possess polarity and are made of multiple subpopulations with different cell cycle parameters. This probably reflects a fundamental principle of organ regeneration, since fin blastema in zebrafish consists of two domains, which are not-dividing *msxb*-expressing distal cells and actively dividing proximal cells [64]. Careful examination of the cell kinetics in diverse regeneration models will clarify this issue.

## 5. Conclusions

The problem of proliferation in animal regeneration from a quantitative perspective has not yet been described in great detail. In this work, we performed an analysis of spatial–temporal proliferation patterns, cell kinetics, and cell sources of regeneration in *A. virens*. Our results show that the annelid regenerative bud is a complex heterogeneous and highly dynamic structure. Understanding how it originates and functions on a cellular level requires finding out how exactly the molecular factors act and how proliferation control is accomplished. Our research on a promising model is an important step towards the comprehensive study of the regeneration phenomenon. In addition to describing the particular quantitative and qualitative characteristics of proliferation, our work raises questions about the general principles of regenerating tissue organization and its regulatory factors. For further progress in these issues, first, complex studies on non-standard organismal models are required, which would allow for comparative analysis at the tissue, cellular, and molecular levels.

## Data Availability

The data presented in this study are available in the Supplementary Materials.

## Supplementary Materials

The following supporting information can be downloaded at https://www.mdpi.com/article/10.3390/cells12101354/s1, File S1: Primary data on the number of labeled cells and the total number of counted cells in each experiment, mean LI values, standard error, and sample size.
